# A multifunctional bioactive material that stimulates osteogenesis and promotes the vascularization bone marrow stem cells and their resistance to bacterial infection

**DOI:** 10.1371/journal.pone.0172499

**Published:** 2017-03-30

**Authors:** Chuang Ma, Qin Wei, Bo Cao, Xinchun Cheng, Juling Tian, Hongwei Pu, Aihemaitijiang Yusufu, Li Cao

**Affiliations:** 1 Department of Orthopedics Center, First Affiliated Hospital of Xinjiang Medical University, Urumqi, China; 2 Department of Orthopedics Center, First Affiliated Hospital of Xinjiang Medical University Chang Ji Branch, Chang Ji, China; 3 Xinjiang Key Laboratory of Medical Animal Model Research, Clinical Medical Research Institute of the First Affiliated Hospital of Xinjiang Medical University, Urumqi, China; 4 Carders Health Care No. 4 Department of Xinjiang Uygur Autonomous Region People's Hospital, Urumqi, China; 5 Department of Clinical Laboratory, The first people's Hospital of Urumqi, Urumqi, China; 6 Department of Science and Research Education Center, First Affiliated Hospital of Xinjiang Medical University, Urumqi, China; Helsingin Yliopisto, FINLAND

## Abstract

The main limitation of tissue engineering lies in the inability to stimulate osteogenesis, angiogenesis of stem cells and broad-spectrum antimicrobial activity. However, the development of multifunctional bioactive materials with these capabilities remains a great challenge. In this study, we prepared mesoporous silica nanoparticles encapsulated with silver nanocrystals (AG-MSN) with uniform sphere size and mesopores. Platelet-derived growth factor BB (PDGF-BB) was effectively loaded in the AG-MSN mesopores (P-AG-MSN). The silicon ions (Si) released by P-AG-MSN stimulate osteogenic differentiation of bone marrow stromal cells (BMSC) by activating the alkaline phosphatase (ALP) activity of bone-related genes and increasing protein (OCN, RUNX2 and OPN) expression. Ag+ ions could be slowly released from the interior of the shell, highlighting their durable antibacterial activity. The sustained release of PDGF-BB from P-AG-MSN stimulated the angiogenic differentiation of BMSC, as indicated by the enhanced secretion of vascular endothelial growth factor (VEGF), HIF-1α, HGF and ANG-1 and protein expression. Our results show that P-AG-MSN can clearly promote BMSC osteostimulation and vascularization. This research serves as a preliminary study of the utilization of this multifunctional mixture to fabricate a new active biological scaffold that integrates BMSC osteostimulation, vascularization and bactericidal effects by 3D printing technology.

## Introduction

The clinical treatment of large segmental bone defects after splintered fracture, tumor resection, or cleaning of osteomyelitis lesions remains a great challenge for orthopedists. Serious bone defects caused by high-energy trauma are usually accompanied by infection in the bone defect site, which blocks normal bone healing, resulting in the formation of osteomyelitis. Consequently, necrosis of the bone, cavity, and sinus tract, among others, further aggravates the severity of the bone defect. Most available bone-repair materials exhibit common problems, such as inadequate biological activity, a slow repair rate, a poor repair effect, and an inability to guard against bacterial infection. Therefore, over the past several years, the treatment of bone defects, including those caused by trauma, tumor, infection or genetic malformation, using multifunctional bioactive materials has attracted extensive attention.[1–3] To repair large segmental bone defects, this biological material should feature properties that include osteostimulation (promoting new bone formation), angiostimulation (inducing vascularization) and the capacity to provide defense against bacterial infection.[4–7] Unfortunately, few synthetic scaffold materials can satisfy all of these properties simultaneously. Most recent research has focused on how to optimize the chemical compositions to enhance the cellular reaction of the biomaterials,[8] i.e., the influence of ions such as Sr, Mg, Zn and Cu on the stimulation of osteoblast and angiogenic differentiation.[3] In many fracture or bone-defect cases, patients usually exhibit inflammation.[9] One extremely key problem in using tissue engineering to promote the bone-healing process is to effectively control inflammation and promote tissue vascularization. An insufficient blood supply to the bone tissue engineered during the initial graft period would block the nutrient supply and excretion of metabolic products, disturbing the signal transmission among cells and destabilizing the intercellular environment, affecting the regeneration of bone tissues.[10,11] Currently, a relatively promising strategy for the vascularized tissue engineering of bone is to jointly culture cells with angioblastic ability and multipotential stem cells to establish vascularized tissue engineering bone.[12] To achieve the multi-functional properties of bone-repairing materials, we propose a single biomaterial system that can induce the multi-directional differentiation of mesenchymal stem cells and provide effective infection control.

In 1970, Carlisle’s research revealed that silicon ions (Si) may play a crucial role in the mineralization process of preosseous tissue.[13] A number of silicate bioactive materials, including Si-substituted calcium phosphates and bioactive glass, have been applied in studies related to osteogenesis.[14]

Mesoporous silica nanoparticles (MSNs) are a new nanoparticle material developed by R. Nooney et al. at the beginning of this century and are characterized by (1) a small size and high body surface area; (2) uniform pore canal distribution and controllable pore diameter size; (3) large pore volume; and (4) large amounts of Si hydroxyl groups distributed on the surface that are capable being modified, thus providing a very promising controllable drug delivery system.[15,16] As a type of silicate-based bioactive material, it is assumed that Si can be used as a drug carrier with osteostimulatory properties.

Silver is a broad-spectrum antibacterial material and is a popular research topic as an inorganic antibacterial agent. Silver has strong bactericidal ability against gram-positive bacteria, gram-negative bacteria and anaerobic bacteria. In recent years, further research has found that MSNs may be loaded with silver ions or silver nanoparticles to manifest its antibacterial function. Jeffrey I. Zink at UCLA prepared ore-shell MCM-41 mesoporous nanoparticles, namely, silver-loaded nanoparticles, and confirmed their effective broad-spectrum antibacterial performance in both liquid-phase suspension medium and LB template.[17]

In recent years, research related to the application of mesoporous silica nanoparticles encapsulated with silver nanocrystals, AG-MSN, in bone have gained substantial attention. However, most studies have concentrated on the improvement of mechanical properties and the bactericidal effects of the polymer/mesoporous silica composite material.[17] These studies revealed a good biocompatibility of AG-MSN; however, they failed to address the bone formation effect of AG-MSN itself.

Platelet-derived growth factor BB (PDGF-BB) is a polypeptide growth factor that is present in many tissues and participates in bone formation as well as bone resorption processes, playing an important role in adjusting bone tissue reconstruction.[18] PDGF-BB exerts essential regulatory effects on fracture healing by enhancing cell proliferation and differentiation in the fracture zone to promote local blood vessel formation.[19,20] Hui Xie et al. found that during bone modeling and remodeling, PDGF-BB secreted by preosteoclasts plays an important role in inducing CD31^hi^Emcn^hi^ blood to form vessel bone modeling and remodeling. The serum and bone marrow levels of PDGF-BB and the numbers of CD31^hi^Emcn^hi^ vessels are notably lower than those in sham-operated controls in an ovariectomy (OVX)-induced osteoporotic mouse model. Exogenous PDGF-BB treatment or cathepsin K inhibition increases the number of preosteoclasts and the endogenous levels of PDGF-BB, consequently increasing the CD31^hi^Emcn^hi^ vessel number and stimulating bone formation in OVX mice.[21]

Apart from the provision of an appropriate surface for cells and the storage and release functions of the compounds, the biomaterial should be capable of promoting vascularization. Successfully immobilizing proteins or peptides on biomaterials in tissue engineering a key tactic to enhance neovascularization events.[22] AG-MSN contain a large volume of space and are, usually used as a carrier for drugs or growth factors.[23] AG-MSN as a carrier represents a new strategy for tissue engineering vascularization because it can gradually release growth factors to promote tissue vascularization and thus promote stem cell vascularization during bone modeling and remodeling. The aims of this study were to explore the BMSC osteostimulation effects of Si, the BMSC angiostimulation capacity of PDGF-BB, and the silver ion bactericidal effect of AG-MSN multifunctional compounds carrying PDGF-BB on bone modeling and remodeling. Additionally, a completely new active biological scaffold was constructed that integrated the BMSC osteostimulation effect, vascularization and bactericidal properties using this multifunctional compound together with 3D print technology. The present findings are intended to propose a new concept for bone tissue regrowth materials, i.e., a single biomaterial system that can induce the multi-directional differentiation of mesenchymal stem cells and also provide effective infection control.

## Materials and methods

This study was carried out in strict accordance with the recommendations in the Guide for the Care and Use of Laboratory Animals of the National Institutes of Health. The protocol was approved by the Committee on the medical ethics committee of the First AffiliatedHospital of Xinjiang Medical University (Ethical appraval number: 20160926–01). In thecourse of the study, the researchers comply with the declaration of biomedical research approach (Trial) with GDP and other relevant ethical principles, ethical standards and related laws, regulations, practices, system. all efforts were made to minimize suffering.

### Synthesis of AG-MSNs

Monodispersed hollow mesoporous silica microspheres with a particle size of approximately 3 μm featuring regular spheres and uniform particle sizes were prepared using a technology that combines the template method and the Stöber method. Ag^+^ was absorbed on the microsphere surface of a sulfonated polystyrene (PS) template by electrostatic attraction and then reduced and stabilized by PVP, thus generating PS/Ag composite microspheres. The surface of the PS/Ag composite microsphere was coated with silica colloidal particles generated from TEOS under homogeneous catalysis action, forming a spherical silica shell. Finally, the PS sphere and PVP were removed by calcining, providing silver-loaded silica microspheres with a hollow mesoporous structure.

### Growth factors related to the loading and release of PDGF-BB in MSNs

AG-MSN (0.8 g) was suspended in 1 mL of phosphate-buffered solution (PBS, pH = 7.4) with 400 μg/200 μg/100 μg of PDGF-BB (Sigma–Aldrich Co. USA) to load the drug molecules into the particle pores. PDGF-BB on the surface of the drug-loaded AG-MSN was cleared by PBS. The AG-MSN compound was prepared upon vacuum dryness after placement at room temperature for 30 min and then frozen at -2°C. After PBS dilution at 37°C, PDGF-BB solutions with different concentrations were derived (400 μg mL^-1^, 200 μg mL^-1^ and 100 μg mL^-1^). The release of PDGF-BB was assessed in an in vitro experiment using a double antibody two-step enzyme-linked immunosorbent assay. Five pieces of the compound were placed in 100 mL of PBS and then placed at 37°C. The changes in PDGF-BB contents in the buffer solution were detected according to the PDGF-BB ELISA kit instructions. In vitro release curves were drawn over the course of the release time as horizontal coordinates, and the cumulative release percent was presented as the vertical coordinate. The AG-MSN loaded with PDGF-BB were immersed into PBS solution at 37°C on a shaker with the shaking frequency of 100 r/min. The release medium was collected at defined time intervals and replaced with fresh PBS solution. The released PDGF-BB from P-AG-MSN was monitored by UV–Vis analysis at 250 nm.

### Detection of silver ion release curves

The 2 mg of P-Ag-MSN was precisely weighed and blended in l0 mL of SBF for 10 min of ultrasonic exposure using an ultrasonic oscillator, resulting in 200 μg/mL SBF. Next, 100, 50, 25, and 12.5 ng/mL SBF were derived using a multiple gradient dilution method. SBF in all groups was divided into 9 EP tubes for storage at 36°C, in a volume of 1 mL per tube. After 30 min, 1 h, 3 h, 6 h, 12 h, 24 h, 3 d, 7 d, l0 d and 14 d, one tube from each group was placed in a high-speed centrifuge for 10 min (7000R centrifuge), and the supernatant was collected. The silver ion concentration in the supernatant was detected using flame atomic absorption spectrometry, which was used to derive the silver ion release curves for all groups.

### Isolation of rat BMSC

All research involving animals was approved by the First Affiliated Hospital of Xinjiang Medical University. Ten healthy SD rats, weighing approximately 150–200 g, were intraperitoneally injected with 30 mg/kg pentobarbital sodium (3% concentration) for general anesthesia. For disinfection, we used a bone marrow puncture needle to puncture the medullary cavity of the fetal bone, which was then connected to a 5-cm injector for medical purposes to aspirate 1–3 mL of the rat marrow (injector must be pre-loaded with anticoagulant heparin sodium). The aspirated marrow was added to a large centrifuge tube containing 3 mL of PBS, which was supplemented with 3 mL of lymphocyte isolation solution for leukomonocyte isolation at room temperature for 3–5 min. Subsequently, it was placed in a super-magnum centrifuge for 10 min (2000 r/min). After centrifugation, the tube was gently removed, and the bone marrow stromal cells located in the middle tier were aspirated into a 5-mL centrifuge tube, washed three times with PBS, inoculated into a 25-mL culture flask at a density of 2x10^4^/cm^2^, and added to 3 mL of DMEM culture medium containing 12% fetal calf serum (l00 U/mL penicillin and l00 mg/L streptomycin with a pH ranging from 7.2–7.4).

### Detection of cellular toxicity

A cytotoxicity test kit (CCK8) was used. BMSC at a certain density were inoculated into MEM culture medium for 24 h and incubated until dissolution of the cells and successful adherence to the culture flask and plate with greater than 80% fusion. Cells grown to 95% confluency were digested into single cells with 0.25% trypsin adjusted to 1x10^5^ pcs/mL The cells were inoculated into a 96-well plate and supplemented with different concentrations of AG-MSN during their growth to 90% confluency. In each well of the 96-well plate, l00-μL cell suspensions were added (approximately 1x10^4^ cells) at 37°C and incubated with 5% CO_2_ for 24 h. The original culture was discarded. The above prepared suspensions for the experimental and control groups were cultured together, and the culture plate was removed on days 1, 3, and 7; the culture solution was discarded, and the cells were washed once with PBS. Ten microliters of the new cellular proliferation and toxicity testing solution was then added for a 4-h culture at 37°C in the presence of 5% CO2. The optical density of every well was determined at a wavelength of 450 nm using a microplate reader, and 3 replicate wells were assessed at each time points in every group. Cellular toxicity was evaluated by comparing the OD values in the experimental and control groups.

### Secretion levels of factors related to osteoblast differentiation

BMSC were inoculated at a specific density with MEM culture medium in the culture plate for 24 h. The cells were successfully adhered to the walls in culture solution or in culture plates and fused at a level exceeding 80%. Subsequently, 0.25% EDTA was added for dissolution, and culture medium was added to a cell suspension of approximately 1x10^5^ cells. With the elimination of 6-well plates, 1-mL cell suspensions and 1 mL of blank culture medium were added to every plate well (approximately 1x10^4^ cells) at 37°C and incubated at 5% CO_2_ for 24 h. The original culture was discarded. The medium suspensions in the experimental and control groups were added for co-culturing, and the culture plate was removed on days 1, 3, 5 and 7. The supernatant was carefully collected and placed in EP pipes, and the OD values of all pores were detected at 450 nm using a spectrophotometer according to the alkaline phosphatase (ALP), OCN and RUNX2 ELISA kits.

### Determination of gene expression related to osteoblast and angioblast differentiation

Total RNA was extracted with TriPure Isolation Reagent (Roche Applied Science, Mannheim, Germany), and 500 ng of total RNA was reverse-transcribed using the Primescript RT reagent kit (Takara Bio, Tokyo, Japan) according to the manufacturer’s protocol. Quantitative real-time PCR for mRNA quantification was performed using SYBR Premix Ex Taq (Takara Bio, Tokyo, Japan) and an Applied Biosystems 7500 Fast Real-Time PCR System (Applied Biosystems, Carlsbad, CA, USA). The expression levels of target genes were normalized to the expression of GAPDH. The following specific forward and reverse primers were used: Five hundred nanograms of total RNA extracted with TriPure Isolation Reagent (Roche Applied Science, Mannheim, Germany) was reverse-transcribed using the Primescript RT reagent kit (Takara Bio, Tokyo, Japan) according to the manufacturer’s instructions. SYBR Premix Ex Taq (Takara Bio, Tokyo, Japan) and an Applied Biosystem 7500 Fast Real-Time PCR System (Applied Biosystems, Carlsbad, CA, USA) were used for mRNA quantification. The following specific forward and reverse primers were used:

ALP: forward, 5'- CCTCCTCGGAAGACACTCTGACC and reverse, 5'-CGCCTGGTAGTTGTTGTGAGCATAG;

OCN: forward, 5'-GCAAAGGTGCAGCCTTTGTG and reverse, 5'-GGCTCCCAGCCATTGATACAG;

OPN: forward, 5'-TCACCTGTGCCATACCAGTTAA and reverse, 5'-TGAGATGGGTCAGGGTTTAGC;

RUNX2: forward, 5'-GTGATAAATTCAGAAGGGAGG and reverse, 5'-CTTTTGCTAATGCTTCGTGT;

VEGF: forward, GCTGTCTTGGGTGCATTGG and reverse, 5'-GCAGCCTGGGACCACTTG;

Ang-1: forward, TGGAGATAGGAACCAGCCTCT and reverse, 5'- TGGATTTCAAGACGGGATGT;

Hgf: forward, 5'-TCGTTCCTTGGGATTATTGC and reverse, 5'-TCGCAGTTGTTTTGTTTTGG; and

GAPDH: forward, 5'-TCAGCAATGCCTCCTGCAC and reverse, 5'-TCTGGGTGGCAGTGATGGC.

All procedures were subject to the instruction provided with the SYBR Premix Ex Taq^TM^ II Kit (TAKARA). The mRNA expression levels of ALP and OC in osteoblasts in the experimental and control groups were determined using the Rotor-Gene3000A fluorescence ratio PCR instrument. Considering GAPDH as an internal reference, the reaction buffer system was prepared. Finally, quantitative detection of gene expression was achieved by analyzing the fluorescence intensity.

### Detection of the expression of key factors

Total soluble protein extracted from cells was resolved on 10% SDS polyacrylamide gels and transferred electrophoretically to nitrocellulose membranes. Blots were blocked with 5% milk followed by an overnight incubation with vascular endothelial growth factor (VEGF) antibody (R&D Systems, 1:300), HFG antibody (Abcam, Cambridge, MA, USA, 1:950), HIF-1α antibody (R&D Systems, 1:500), Ang-1 antibody (R&D Systems, 1:600), and GAPDH antibody (Beyotime, Beijing, China, 1:1000). The blots were then incubated with horseradish peroxidase-labeled goat anti-mouse IgG secondary antibody (Beyotime) and visualized using enhanced chemiluminescence substrate (Beyotime).

Total soluble proteins extracted from cells were resolved on 10% SDS polyacrylamide gels and then electrophoretically transferred to nitrocellulose membranes. After an overnight incubation with VEGF antibody (R&D Systems, 1:300), HFG antibody (Abcam, 1:950), HIF-1α antibody (R&D Systems, 1:500), Ang-1 antibody (R&D Systems, 1:600) and GAPDH antibody (Beyotime, Beijing, China, 1:1000), the blots were blocked with 5% milk, incubated with horseradish peroxidase-labeled goat anti-mouse IgG secondary antibody (Beyotime), and finally visualized using enhanced chemiluminescence substrate (Beyotime).

### Determination of the influence of P-AG-MSN on inflammation

#### Preparation of bacterial suspensions of standard strains

The standard adopted strains (Escherichia coli ATCC25922, Staphylococcus aureus ATCC25923, Pseudomonas aeruginosa ATCC27853, Candida sporogenes ATCC90029, Bacteroides fragilis ATCC25285) were provided by the clinical laboratory of the First Affiliated Hospital of Xinjiang Medical University. The standard concentrates for the above strains were coated on blood agar plates (Escherichia coli, Staphylococcus aureus, Pseudomonas aeruginosa) or salmonella Lowenstein-Jensen (Candida sporogenes) in a 36°C incubator for 18–24 h for cell resuscitation (Candida sporogenes requires a longer time of approximately 24–36 h). The duration of bacterial growth throughout the coating area was determined by the time at which the bacteria reached the logarithmic phase. Single colonies were picked from the plate with an aseptic cotton bud and dissolved in 5 mL of normal saline up to 0.5 turbidity using standard turbidimetry, resulting in a bacterial suspension of 1.5x10^8^ pcs/L which was diluted to 1x10^6^ pcs/L via a 150-fold dilution (l00 μL of bacterial liquid dissolved in 15 mL of normal saline).

#### Experimental procedures to detect materials, MIC and MBC

Six small test tubes were marked as the different groups. The control groups that did not contain silver material were generated by adding 1 mL of pure culture medium to tube No. 1 along with 1 mL of medium suspension containing 100 μg/mL P-AG-MSN into tube No. 2. The experimental groups were generated by adding 1 mL of medium suspension containing 100, 50, and 25/12.5 U μg/mL P-AG-MSN to tubes No. 3 to No. 6. Then, 1 mL of 1x10^6^ pcs/L of the target bacterial suspension was added to 6 tubes, and thus, each mixed system contained 5x10^5^ pcs/L bacteria. The No. 1 tube was considered the bacterial control group, the No. 2 tube as the material control group, and the No. 3 to No. 6 tubes as the experimental groups containing 100, 50, 25, and 12.5 μg/mL P-Ag-MSN, respectively. The tubes were placed in a 36°C bacteriological incubator for 24 h and then removed for observation. A comparison of the experimental groups and bacterial control groups revealed that the lowest density of clear suspension was the MIC of the material against the bacterium. One hundred microliters of mixed liquid collected from every tube were coated on the plate, which was then placed in a 36°C bacteriological incubator for 24 h and finally removed to observe and count the bacterial colonies. The concentration of material in the experimental groups without bacterial growth or a colony number less than 5 represented the MBC of the material against this bacterium.

#### Experimental procedures to detect the OD600 bacterial growth curve

Using an aseptic 96-well plate, the middle 6 rows were marked on the plate cover as the experimental area. Material suspensions of bacteria in all groups were transferred into the 96-well plate with 200 μL added to every well. Using 4 wells per experimental group and a 5^th^ well containing 100 μL of material suspension from the group and l00 μL of pure culture medium, which was set as zero. After 0, 3, 6, 9, 12, 18, and 24 h of culture in an incubator, every well of the plate was removed, blown evenly, and then evaluated using a spectrophotometer. The absorption peak values were detected at OD600, and bacterial growth curves were drawn based on the average values of 4 wells in all groups after adjustment to zero for the detected data.

### Morphological observation of cells

After P-AG-MS induction, on days 2, 4 and 7, cellular morphology and growth were observed using a confocal laser-scanning microscope. At each time point, 3 images were obtained, and each image consisted of 4 views.

### Statistical methods

SPSS 17.0 statistical software was used for the statistical analysis of the data. The data are represented by the mean±standard deviation (X±S), and they were analyzed by one-way ANOVA or two-way ANOVA. P<0.05 was regarded as a significant difference.

## Results

### The morphology and structure of P-AG-MSNs

**Fig 1:**
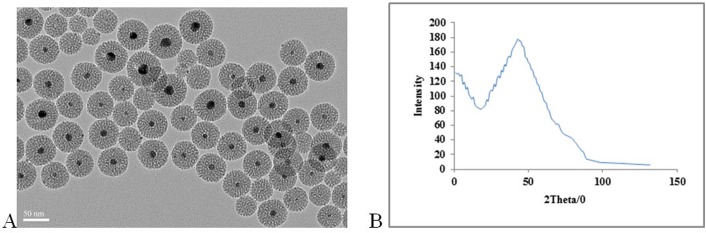
TEM images and small-angle XRD. TEM images and small-angle XRD analysis for PDGF-BB -AG-MSN. (A) TEM image, and (B) small-angle XRD pattern.

The dispersity and morphology of the MSNs were observed by TEM (Fig 1).

The TEM image revealed the dispersed nature and morphology of MSN (Fig 1).

The low-magnification TEM analysis showed that the size of the spheres was predominantly between 30–40 nm. The small-angle X-ray diffraction (SAXRD) analysis revealed a distinct diffraction peak at 2θ = 2°, suggesting that the MSNs had an ordered mesostructure.

From low-magnification TEM analysis, the size of the spheres was typically between 30–40 nm. A distinct diffraction peak observed at 2θ = 2° was displayed by small-angle X-ray diffraction (SAXRD) analysis, suggesting an ordered mesostructure of the MSNs (Fig 1).

### Detection of the silver ion release curve of P-Ag-MSNs in simulated body fluid

**Fig 2:**
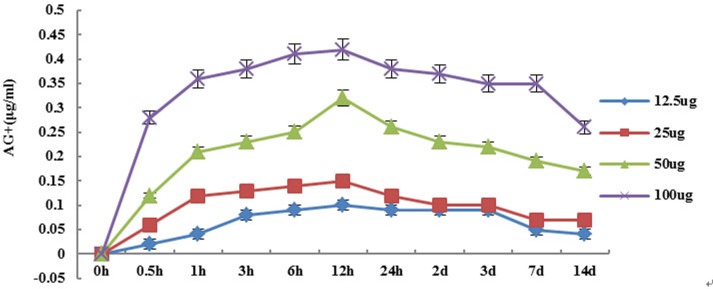
Silver ion release curve of P-AG-MSN. Silver ion release curve of P-AG-MSN with different concentrations in simulated body fluid.

The release of silver ions peaked after soaking the P-AG-MSNfor 3–6 h, with the silver ion release content accounting for 30% of the total silver content, and the content of silver ion gradually decreasing after 3 d of soaking (Fig 2). During the whole release process, the overall release trends in all concentration groups were basically equivalent.

### PDGF-BB loading and release behavior of MSNs

**Fig 3:**
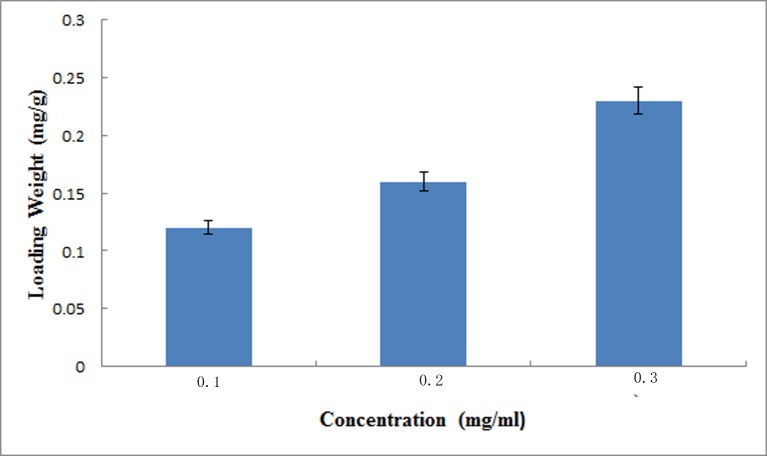
Loading weight of PDGF-BB in AG-MSN. Loading weight of PDGF-BB in AG-MSN that soaked in PBS solution with different PDGF-BB concentrations. With the increasing concentration of PDGF-BB in PBS solution, the loading amount of PDGF-BB also increased.

**Fig 4:**
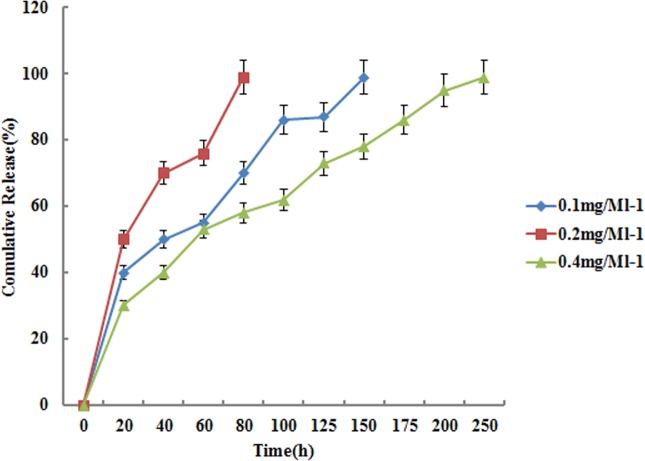
Cumulative release profile of P-AG-MSN in PBS solution. The 70–80% burst release of loaded P-AG-MSN during the first 3 days and the drug release of 3P-AG-MSN were sustained up to day 10.

With the increasing concentration of PDGF-BB in PBS solution, the loading amount of PDGF-BB also increased (Fig 3). The diffusion of drug molecules was controlled by the concentration gradients between the external solution and mesopore channels, and the main force for drug loading was physical adsorption. Thus, drug molecules varied from increasing concentrations of PDGF-BB solutions. The zeta-potential test revealed that the absolute value of nanospheres increased from 8.32 to 18.73 (Table 1) after loading with increasing amounts of PDGF-BB. These results demonstrated successful loading of PDGF-BB in mesopores. During the whole release process, the overall release trends of all concentration groups were basically identical. The 70–80% burst release of loaded P-AG-MSN during the first 3 days and the drug release of 3P-AG-MSN were sustained up to day 10 (Fig 4).

**Table 1:** Zeta-potential of AG-MSN and P-AG-MSN. 1D-MSN, 2D-MSN and 3D-MSN, represented different loaded concentrations of MSN loaded at different concentrations (1 mg mL^-1^, 2 mg mL^-1^ and 3 mg mL^-1^) in terms of the P-AG-MSN-PBS solutions.

	Day 1	Day 3	Day 5	Day 7
Con	100%	100%	100%	100%
P-AG-MSN 6.25	105.43%	101.23%	95.76%	94.86%
P-AG-MSN 12.5	102.58%	97.65%	94.85%	93.69%
P-AG-MSN 25	96.75%	95.82%	93.77%	92.65%
P-AG-MSN 50	89.76%	87.61%	89.67%	88.53%

### Detection of material cytotoxicity (MG63) using the CCK8 method

**Fig 5:**
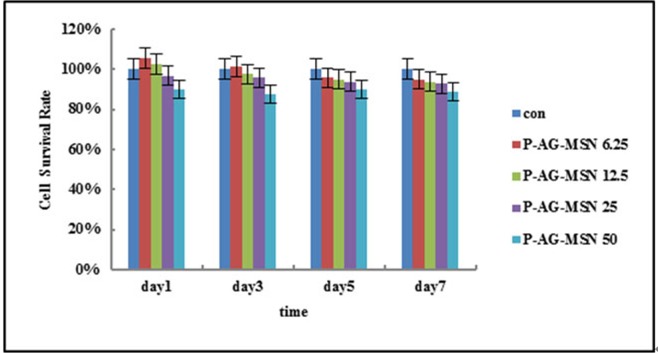
Effect of P-AG-MSNon the metabolic activity of BMSC. P-AG-MSN at 50 μg/m had a slight inhibitory effect on BMSC growth capacity; however, in the 4 P-AG-MSN groups, the 4 different concentrations did not inhibit BMSC growth, with insignificant findings on days 3, 5 and 7.

The relative growth ratio (RGR) was calculated based on the OD value determined using the CCK8 method. P-AG-MSN at 50 μg/m had a slight inhibitory effect on BMSC growth capacity; however, in the 4 P-AG-MSN groups, the 4 different concentrations did not inhibit BMSC growth, with insignificant findings on days 3, 5 and 7 (Table 2, Fig 5).

**Table 2:** The effect of P-Ag-MSN on the metabolic activity of BMSC.

Particles	P-AG-MSN	1P-AG-MSN	2P-AG-MSN	3P-AG-MSN
Zeta-potential (mv)	-8.32	-10.23	-12.65	-18.73

### Expression of bone-related genes in BMSCs cultured with P-AG-MSNs

**Fig 6:**
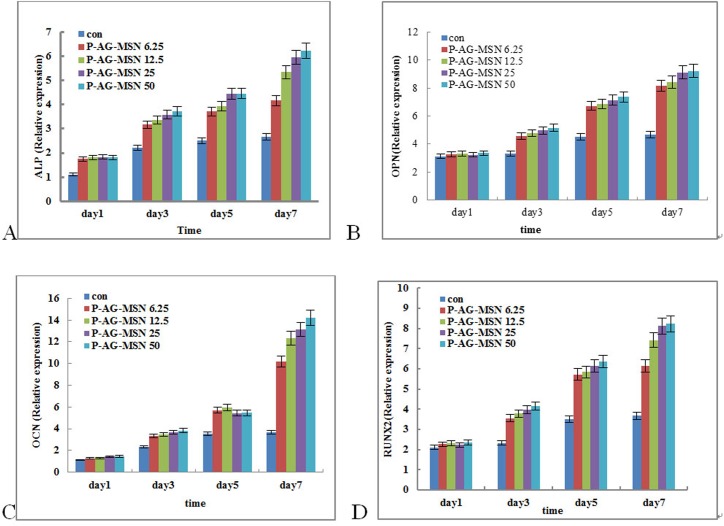
Effect of P-AG-MSN on bone-related gene expression in BMSC after 7 days of culture. (A) ALP, (B) OPN, (C) OCN and (D) RUNX2. (Comparison between P-AG-MSN group and blank control, comparison between MSN and D-MSN groups, P<0.05).

**Fig 7:**
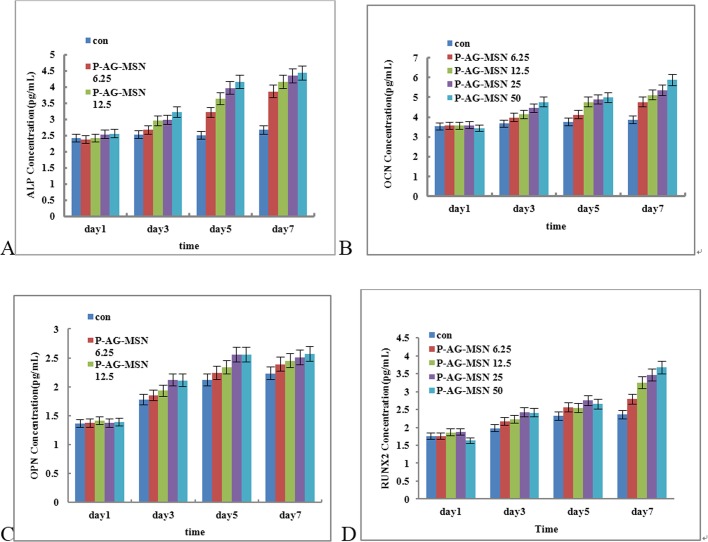
ELISA analysis of ALP, OCP and RUNX2 protein expression in BMSC cultured with different concentrations of AG-MSN and P-AG-MSN for 7 days. (A) ALP, (B) OPN, (C) OCN and (D) RUNX2 (comparison between the P-AG-MSN group and blank controls, comparison between MSN and D-MSN groups, P<0.05).

**Fig 8:**
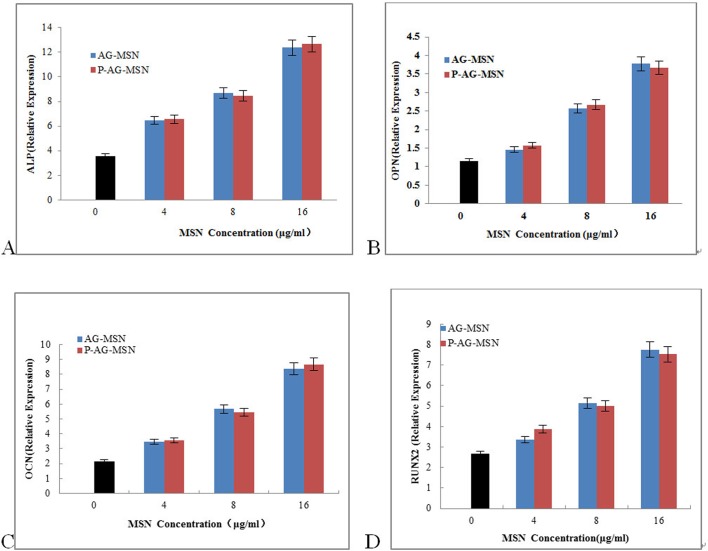
The effect of AG-MSN and P-AG-MSN on bone-related gene expression of BMSC after 7 days of culture. (A) ALP, (B) OPN, (C) OCN and (D) RUNX2 (comparison between AG-MSN and P-AG-MSN groups, P>0.05).

ALP gene expression levels at different time points in all experimental groups and control groups were detected by real-time qRT-PCR. The table shows the comparison of all experimental groups with control groups. The ALP gene expression quantities were not significantly different; however, the ALP gene expression levels in the P-AG-MSN group of osteoblasts were significantly increased compared with the control groups, reaching significance (* versus Con, P<0.05; one-way ANOVA). The results further revealed that the expression levels of OCN, OPN and RUNX2 were increased depending on nanosphere concentration, especially the P-AG-MSN concentration (Fig 6B, 6C and 6D. ELISA analysis has shown that the expression of OCN, OPN and RUNX2 proteins is consistent with the real-time qRT-PCR results (Fig 7). However, an equivalent concentration of AG-MSN and P-AG-MSN showed no significant difference (Fig 8).

### Capillary network formation after BMSC are induced by P-AG-MSN

**Fig 9:**
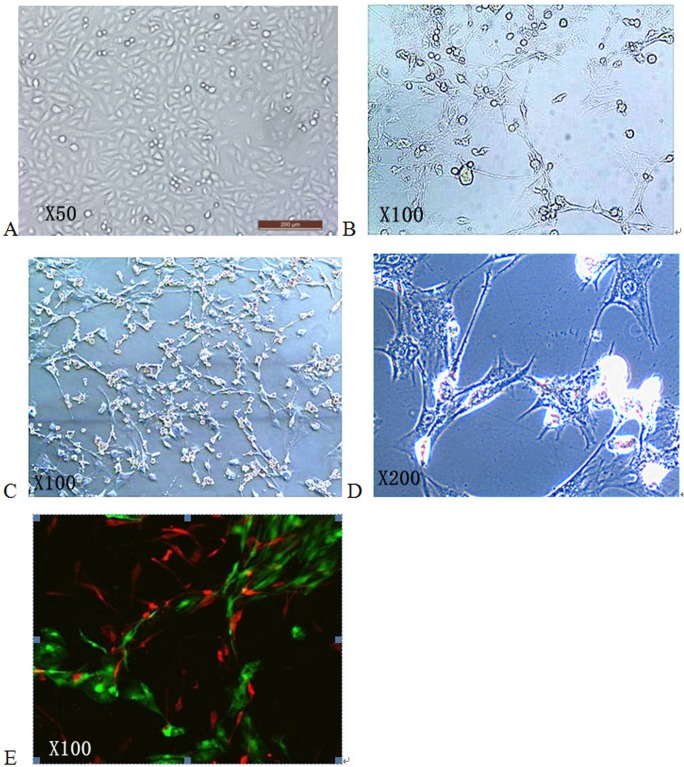
The effect of P-AG-MSN on angiogenic differentiation in BMSC. After 7 days, the BMSC displayed an obvious stretch and gathered to form a vessel-like reticular structure that gathered, closed up, and even participated in the process.

Laser scanning confocal microscopy was adopted for observation after culture for 2, 4 and 7 days. After 2 days, the BMSC showed no obvious gathering trend, exhibited slightly stretched small cell parts and showed no interaction or contact between two types of cells (Fig 2A). After 4 days, most BMSC took on an apparent gathering and tensile trend, with interconnections between cells and a visible mild tubular structure. After 7 days, the BMSC displayed an obvious stretch and gathered to form a vessel-like reticular structure that gathered, closed up, and even participated in the process (Fig 9). The tubular structure was obvious, most of which was formed by BMSC. BMSC were the major elements of the microvessels forming the co-culture system, maintaining and stabilizing the vessel-like reticular structure. However, BMSC surrounded the microvessels, and some BMSC functioned together with HUVECs to form the tube wall (Fig 9).

**Fig 10:**
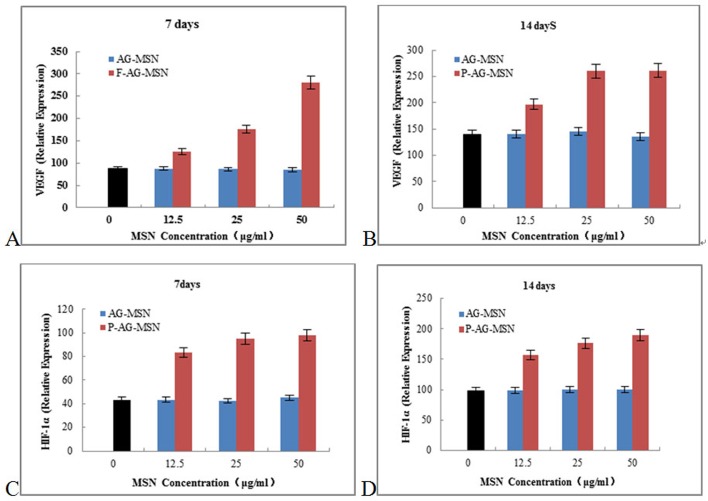
The effect of P-AG-MSN on VEGF, HIF-1α, Ang-1 and HGF gene expression in BMSC. P-AG-MSN was added, VEGF secreted from BMSC clearly increased, and the gene expressions levels of HIF-1α, HGF and Ang-1 also increased following the addition of P-AG-MSN to medium. (A, B) VEGF,(C, D) HIF-1α.

Fig 10 shows that as P-AG-MSN was added, VEGF secreted from BMSC clearly increased, and the gene expressions levels of HIF-1α, HGF and Ang-1 also increased following the addition of P-AG-MSN to medium.

### The effect of P-AG-MSN on VEGF, HIF-1α, Ang-1 and HGF protein expression in BMSC

**Fig 11:**
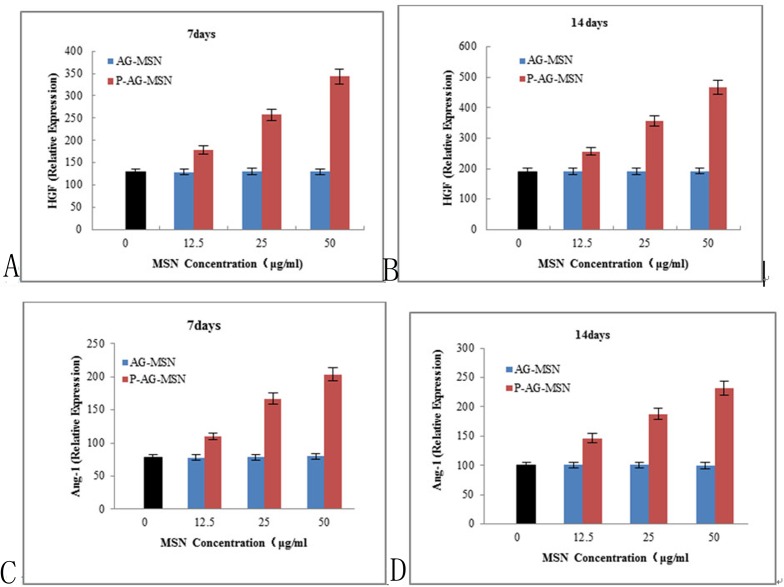
The effect of solutions with different AG-MSN and P-AG-MSN concentrations on gene expression in BMSC on day 7. (A, B) HGF and (C, D) Ang-1.

**Fig 12:**
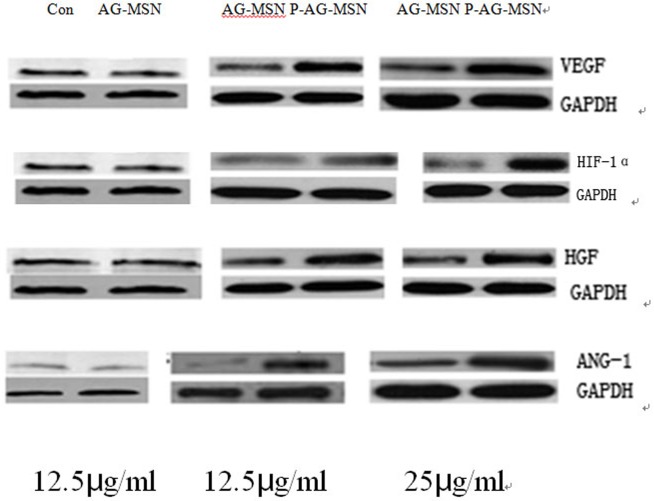
The effect of P-AG-MSN on VEGF, HIF-1α, Ang-1 and HGF protein expression in BMSC. VEGF secreted from BMSC was obviously increased with the addition of P-AG-MSN, and the protein expression of HIF-1α, HGF and Ang-1 also increased following the addition of P-AG-MSN to the medium.

The following experiments were conducted to further explore the effect of P-AG-MSNs on the angiogenic differentiation of BMSC. The protein expression of HIF-1α, HGF and Ang-1 increased following the addition of P-AG-MSN to the medium (Fig 11) and shows that VEGF secreted from BMSC was obviously increased with the addition of P-AG-MSN (Fig 12). In addition, Si did not affect the angiogenic activity of BMSC compared with the control group.

### Detection of P-Ag-MSNs against related pathogenic bacteria

The results showed that the MBCs of P-AG-MSN against Escherichia coli, Pseudomonas aeruginosa and Candida sporogenes were 50/400/100 μg/100 mL, respectively; however, P-AG-MSN exhibited poor bactericidal effects against Staphylococcus aureus. Figs 11 and 12 show the detection of the growth curves of various bacteria, which revealed that 100/50/25 μg/mL of the Escherichia coli group could inhibit bacterial growth in 12 h compared with the control group (P<0.05); 100/50 μg/mL of the Candida sporogene group could remarkably inhibit bacterial growth in 12 h compared with the control group (P<0.05); 100/50 μg/mL of the Pseudomonas aeruginosa group could remarkably inhibit bacterial growth in 12 h compared with the control group (P<0.05); and 100 μg/mL of the Staphylococcus aureus group could remarkably inhibit bacterial growth in 12 h compared with the control group (P<0.05). These results suggested that P-AG-MSN possessed broad-spectrum antibacterial ability toward common pathogenic bacteria of chronic osteomyelitis in vitro.

**Fig 13:**
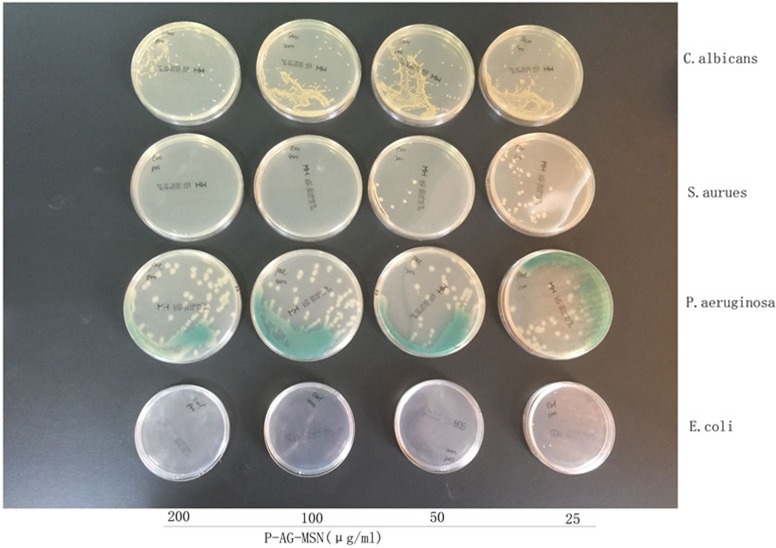
MAC concentrations of Escherichia coli, Pseudomonas aeruginosa, Candida sporogenes, and Bacteroides fragilis. The observed lowest P-AG-MSN material concentration with an asepsis colony or bacterial colony number less than 5 was the lowest bactericidal concentration of this bacterium after all bacterial experimental groups were coated and cultured for 24 h.

Fig 13 shows the MBC detection of materials. The image shows that the observed lowest P-AG-MSN material concentration with an asepsis colony or bacterial colony number less than 5 was the lowest bactericidal concentration of this bacterium after all bacterial experimental groups were coated and cultured for 24 h. The results showed that the MAC concentrations against Escherichia coli, Pseudomonas aeruginosa, Candida sporogenes, and Bacteroides fragilis were 50/400/100 μg/mL; however, P-AG-MSN had no obvious bactericidal effect on Pseudomonas aeruginosa.

**Fig 14:**
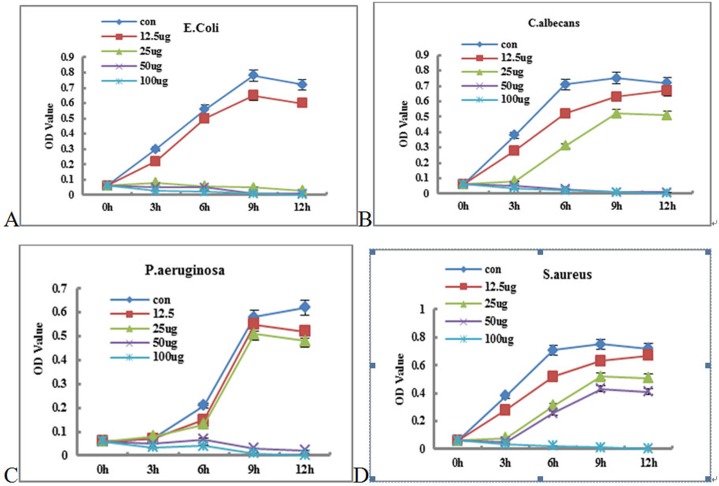
Growth curves of various bacteria. (A) Compared with the control group, 100/50/25 μg/mL Escherichia coli could remarkably inhibit bacterial growth (P<0.05) at 12 h. (B) Compared with the control group, 100/50 μg/ml Candida sporogenes could remarkably inhibit bacterial growth (P<0.05) at 12 h. (C) Compared with the control group, 100/50 μg/mL Pseudomonas aeruginosa could remarkably inhibit bacterial growth (P<0.05) at 12 h. (D) Compared with the control group, 100 μg/mL Staphylococcus aureus could remarkably inhibit bacterial growth (P<0.05).

Fig 14 Detection of the growth curves of various bacteria: (A) Compared with the control group, 100/50/25 μg/mL Escherichia coli could remarkably inhibit bacterial growth (P<0.05) at 12 h. (B) Compared with the control group, 100/50 μg/ml Candida sporogenes could remarkably inhibit bacterial growth (P<0.05) at 12 h. (C) Compared with the control group, 100/50 μg/mL Pseudomonas aeruginosa could remarkably inhibit bacterial growth (P<0.05) at 12 h. (D) Compared with the control group, 100 μg/mL Staphylococcus aureus could remarkably inhibit bacterial growth (P<0.05).

## Discussion

Most bone defects form due to chronic osteomyelitis, which is an inflammatory pathological process that accompanies bone destruction caused by microbial infection. Pathopoiesia of osteomyelitis is usually a mixed infection caused by various bacteria, mostly consisting of Staphylococcus aureus (accounting for 75% of the pathogenic bacteria), followed by fungi and anaerobes such as Escherichia coli, Pseudomonas aeruginosa, Streptococcus hemolyticus, and Candida sporogenes, among others.[24,25] Patients with bone defects must fabricate tissue-engineered bone to repair the affected area after receiving surgical treatment. Thus, there is a growing need for composite tissue engineering materials with good biocompatibility, a certain bone induction ability, vascular induction ability and strong bactericidal ability.[26–28] To achieve this goal, we have systematically studied the facilitation effect of Si on BMSC osteostimulation, the facilitation effect of PDGF-BB on BMSC angiostimulation and the bactericidal effect of silver ions on AG-MSN multifunctional mixtures loaded with PDGF-BB during bone modeling and remodeling.

Presently, many studies have reported that mesoporous oxide nanoparticles have no obvious cytotoxicity toward multiple cell types.[29–31] Our study demonstrated that P-AG-MSN lack obvious cytotoxicity under the appropriate concentration. ALP is a key indicator during the early stage of osteoblastic differentiation, serving as the major functional enzyme during the process of osteoblast mineralization. It is also generally recognized as a major characteristic enzyme of osteoblastic differentiation. OPN and OCN serve as important markers of osteogenic differentiation, while RUNX2 is a master regulator of osteogenesis commitment.[32–34] Studies have shown that these proteins support the formation of bone-like nodules, representing the foundation for further matrix mineralization.[35] The experimental results have shown that the activity of ALP in all experimental groups was not significantly different compared with the control group on day 1. From days 3 to 7, the activities of ALP, CON, CPN and RUNX2 in the P-Ag-M group rose compared with the control group. Thus, to explore the biological effect of Si on the differentiation of human bone mesenchymal stem cell differentiation into osteocytes, we used P-AG-MSN and MSN and blank control groups in the following experiment. The results revealed that Si are the major factor underlying the stimulation of BMSC differentiation into osteocytes.

This compound biomaterial should not only provide an appropriate surface for cells and have storage and release functions, but it should also be capable of promoting vascularization. The enhancement of neovascularization events lies in the successful immobilization of proteins or peptides on biomaterials for tissue engineering. Angiogenic growth factors are necessary powerful initiators for neovascularization,[10] which activate and stimulate endothelial (progenitor) cells to migrate towards the factor gradient and also promote cell assembly, vessel formation and maturation. VEGF is the main factor responsible for upregulating angiogenic processes and is often used to induce neovascularization in engineered tissues. Many difficulties must be overcome to effectively release angiogenic factors. An area-restricted and long-term delivery strategy can be used increasingly as a substitute for bolus injection of growth factors to maintain angiogenic factors in a highly unstable state in vivo.[36] To design new biomaterials to confront the high degradation rate of growth factors is a very promising approach. Effective targeting has been controlled with the use of biomaterials that contain degradable porous reservoir structures or pre-encapsulated microspheres.[37] The body surface area of AG-MSN (462.7 m^2^ g^-1^) herein was relatively large (462.7 m^2^ g^-1^), offering sufficient space for loading PDGF-BB (462.7 m^2^ g^-1^). Loaded PDGF-BB could be gradually released during relapse. Previous studies have shown that preosteoclasts secrete PDGF-BB to recruit EPCs and BMSC, promoting angiogenesis when coupled with osteogenesis. Our study demonstrated that PDGF-BB released from AF-MSN can also obviously promote BMSCsvascularization. It is known that PDGF-BB induces EPC migration and promotes angiogenesis,[38] and differentiation of the microenvironment of osteoprogenitors is affected by vessel formation.[39–41] PDGF-BB released by P-AG-MSN induces the formation of CD31hiEmcnhi vessel subtypes, promoting the coupling of angiogenesis with bone formation. The HIF-1 pathway is an important pathway in the regulation of vascularization.[42,43] HIF-1 is an oxygen-sensitive dimeric complex consisting of two subunits, i.e., HIF-1α and HIF-1β. HIF-1 triggers the activation of VEGF. In our study, the expression levels of VEGF, HGF, Ang-1 and HIF-1α were significantly increased by culturing BMSC with P-AG-MSN compared with MSNs and the blank control groups, revealing that the release of PDGF-BB from MSNs could upregulate the angiogenic differentiation of BMSC. Together, these genetic and pharmacological findings suggest that the stimulation of PDGF-BB released by P-AG-MSN may promote the combination of angiogenesis with bone formation, representing a potential therapeutic strategy for bone defects.

In the past 20 years, the antibacterial action of nanocrystallized silver ions has undergone a qualitative leap. It has been reported that tiny amounts of nanosilver can exert powerful bactericidal effects. The antibacterial properties of silver nanoparticles is far beyond that of traditional silver ion bactericides,[44,45] possessing better bactericidal effect. With a powerful bactericidal effect on gram-positive bacteria, gram-negative bacteria and fungi, silver ions represent veritable broad-spectrum antibacterial agent.[46] Thus, we assume that the treatment of bone defects accompanying multiple compound infection chronic osteomyelitis via the construction of P-AG-MSN tissue engineering scaffolds is very promising.

In this study, we detected in vitro antibacterial properties and the release of silver ion using P-AG-MSN nanoparticles. The results showed that the P-AG-MSNMIC against Staphylococcus aureus, Pseudomonas aeruginosa, Escherichia coli, and Bacteroides fragilis is 200 μg/mL, while the MBC is 400, 200, 200, and 200 μg/mL, respectively, revealing that the silver and silver ion exerted antibacterial activities.

## Conclusion

**Fig 15:**
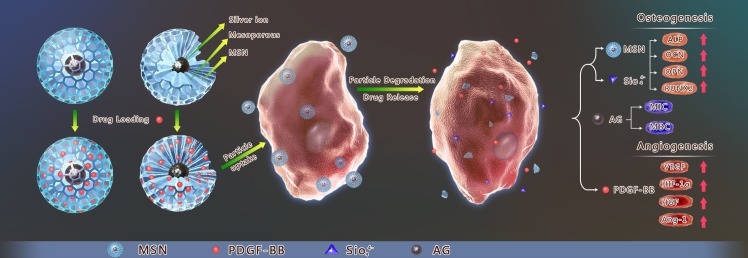
The stimulation of osteogenesis and angiogenesis in bone marrow stromal cells by mesoporous silica nanospheres and PDGF-BB delivery. AG-MSN loaded with PDGF-BB can promote BMSC osteostimulation and vascularization.

Our research showed that AG-MSN loaded with PDGF-BB can clearly promote BMSC osteostimulation and vascularization, and it can serve as a preliminary study for utilizing this multifunctional mixture to fabricate a new active biological scaffold that integrates BMSC osteostimulation and stimulation of vascularization and bactericidal effects using 3D printing technology (Fig 15).
